# Performance of scores in the prediction of clinical outcomes in patients admitted from the emergency service

**DOI:** 10.1590/1518-8345.4722.3479

**Published:** 2021-09-03

**Authors:** Luana Matuella Figueira da Silva, Luciano Passamini Diogo, Letícia Becker Vieira, Fabiano Da Costa Michielin, Michelle Dornelles Santarem, Maria Luiza Paz Machado

**Affiliations:** 1 Hospital Nossa Senhora da Conceição, Centro de Terapia Intensiva, Porto Alegre, RS, Brazil.; 2 Universidade Federal do Rio Grande do Sul, Escola de Enfermagem, Porto Alegre, RS, Brazil.; 3 Hospital de Clínicas de Porto Alegre, Emergência, Porto Alegre, RS, Brazil.

**Keywords:** Sepsis, Septic Shock, Systemic Inflammatory Response Syndrome, Emergency Medical Services, Organ Dysfunction Scores, Nursing, Sepse, Choque Séptico, Síndrome da Resposta Inflamatória Sistêmica, Serviços Médicos de Emergência, Escores de Disfunção Orgânica, Enfermagem, Sepsis, Choque séptico, Síndrome de Respuesta Inflamatoria Sistémica, Servicios Médicos de Urgencia, Puntuaciones en la Disfunción de Órganos, Enfermería

## Abstract

**Objective:**

to evaluate the performance of the quickSOFA scores and Systemic Inflammatory Response Syndrome as predictors of clinical outcomes in patients admitted to an emergency service.

**Method:**

a retrospective cohort study, involving adult clinical patients admitted to the emergency service. Analysis of the ROC curve was performed to assess the prognostic indexes between scores and outcomes of interest. Multivariate analysis used Poisson regression with robust variance, evaluating the relationship between variables with biological plausibility and outcomes.

**Results:**

122 patients were selected, 58.2% developed sepsis. Of these, 44.3% had quickSOFA ≥2 points, 87% developed sepsis, 55.6% septic shock and 38.9% died. In the evaluation of Systemic Inflammatory Response Syndrome, 78.5% obtained results >2 points; of these, 66.3% developed sepsis, 40% septic shock and 29.5% died. quickSOFA ≥2 showed greater specificity for diagnosis of sepsis in 86% of the cases, for septic shock 70% and for mortality 64%, whereas the second score showed better results for sensitivity with diagnosis of sepsis in 87.5%, septic shock in 92.7% and death in 90.3%.

**Conclusion:**

quickSOFA showed by its practicality that it can be used clinically within the emergency services, bringing clinical applicability from the risk classification of patients for the early recognition of unfavorable outcomes.

## Abstract (pt)

**Objetivo:**

avaliar o desempenho dos escores *quick*SOFA e Síndrome da Resposta Inflamatória Sistêmica como fatores preditores de desfechos clínicos em pacientes admitidos em um serviço de emergência.

**Método:**

coorte retrospectiva, envolvendo pacientes adultos clínicos admitidos em serviço de emergência. A análise da curva ROC foi realizada para a avaliação dos índices prognósticos entre escores e desfechos de interesse. Análise multivariável utilizou regressão de Poisson com variância robusta avaliando a relação entre as variáveis com plausibilidade biológica e os desfechos.

**Resultados:**

foram selecionados 122 pacientes, 58,2% desenvolveram sepse. Destes 44,3% tiveram *quick*SOFA ≥2 pontos, 87% desenvolveram sepse, 55,6% choque séptico e 38,9% morreram. Na avaliação de Síndrome da Resposta Inflamatória Sistêmica 78,5% obtiveram resultados >2 pontos, destes 66,3% desenvolveram sepse, 40% choque séptico e 29,5% morreram. O *quick*SOFA ≥2 apresentou maior especificidade para diagnóstico de sepse em 86% dos casos, para choque séptico 70% e para mortalidade 64%, já o segundo escore mostrou melhores resultados para sensibilidade com diagnóstico de sepse de 87,5%, choque séptico 92,7% e óbito 90,3%.

**Conclusão:**

o *quick*SOFA demonstrou pela sua praticidade que pode ser utilizado clinicamente dentro dos serviços de emergência trazendo aplicabilidade clínica a partir da classificação de risco de pacientes para o reconhecimento precoce de desfechos desfavoráveis.

## Abstract (es)

**Objetivo:**

evaluar el rendimiento de los puntajes *quick* SOFA y del Síndrome de Respuesta Inflamatoria Sistémica como predictores de desenlaces clínicos en pacientes ingresados en un servicio de emergencia.

**Método:**

cohorte retrospectiva de pacientes clínicos adultos ingresados en el servicio de emergencia. El análisis de la curva ROC se realizó para evaluar los índices de pronóstico entre puntajes y desenlaces de interés. El análisis multivariado utilizó regresión de Poisson con varianza robusta, evaluando la relación entre las variables con plausibilidad biológica y los desenlaces.

**Resultados:**

se seleccionaron 122 pacientes, 58,2% desarrollaron sepsis. De estos, el 44,3% tenía *quick* SOFA ≥2 puntos, el 87% desarrolló sepsis, el 55,6% *shock* séptico y el 38,9% falleció. En la evaluación del Síndrome de Respuesta Inflamatoria Sistémica el 78,5% obtuvo resultados ≥2 puntos, de los cuales el 66,3% desarrolló sepsis, el 40% *shock* séptico y el 29,5% falleció. El *quick* SOFA ≥2 mostró mayor especificidad para el diagnóstico de sepsis en el 86% de los casos, para *shock* séptico en el 70% y para mortalidad en el 64%, mientras que el segundo puntaje mostró mejores resultados de sensibilidad para el diagnóstico de sepsis de 87,5%, *shock* séptico 92,7% y muerte 90,3%.

**Conclusión:**

el *quick* SOFA demostró, por su practicidad, que se puede utilizar clínicamente dentro de los servicios de emergencia aportando aplicabilidad clínica por medio de la clasificación de riesgo de los pacientes para el reconocimiento temprano de desenlaces desfavorables.

## Introduction

Over the years, the definitions and discoveries about sepsis have been expanding, and in 1991 the concept for sepsis was determined and the use of the SIRS (Systemic Inflammatory Response Syndrome) criterion was introduced, in which four criteria are evaluated: tachycardia, tachypnea fever or hypothermia and leukocytosis or leukopenia^(1)^.

Currently, sepsis is defined by the presence of life-threatening organ dysfunction, secondary to a non-regulated organism response to infection, and is considered a serious disease with a poor prognosis if not treated early. Organ dysfunction is diagnosed by a variation of two points or more in the Sequential Organ Failure Assessment (SOFA) score. Septic shock, on the other hand, is determined as sepsis accompanied by profound circulatory, cellular and metabolic abnormalities capable of substantially increasing mortality compared to isolated sepsis and can be identified in patients who require vasopressors to maintain a stable blood pressure and serum lactate level upon lacking hypovolemia^(2)^.

Sepsis is considered worldwide as a public health problem since it presents high rates of morbidity and mortality. According to studies at the Intensive Care Center (*Centro de Tratamento Intensivo*, CTI), Brazil has come to present rates of more than 200,000 deaths *per* year of users who have received treatment^(3)^.

In addition to that, it has a substantial impact on the health costs. A Brazilian study carried out with adult septic patients admitted to an intensive care center in a public hospital described that the median total cost of sepsis treatment was US$ 9,632,000, with a daily value *per* patient of US$ 934^(4)^.

It is also worth mentioning that the quality of life and cognitive function of sepsis survivors can be permanently impaired^(5)^. The main interventions to improve results in this population of ill patients include early recognition and initiation of adequate therapy, especially with broadspectrum antibiotics and fluid therapy^(6-7)^.

To facilitate the identification of patients with infection and with greater likelihood of unfavorable outcomes, the score called quickSOFA (qSOFA) is used, which is a tool of rapid applicability that can be used at the patient’s bedside and serves as an “alert”, being positive for a possible diagnosis of sepsis, when showing two or more criteria (points) for patient evaluation^(2)^. A previously conducted study mentions that approximately 50% of the patients with severe sepsis receive first aid in emergency services^(8)^.

In view of this magnitude, easy-to-apply tools in the initial approach to the patient become a facilitator in the identification of diseases such as sepsis, with qSOFA and SIRS being viable alternatives for this objective^(2)^. Against this background, the multiprofessional team must prioritize an early diagnosis that can adapt the time necessary for the initiation of the recommended treatment. This first contact with the diagnosis often ends up being in the emergency services. It is known that the overcrowding of these sectors, in view of the increased search for users with signs and symptoms related to sepsis, contributes and brings up the need to identify predictors with high mortality rates, such as this one, in addition to other clinical outcomes.

Even with the increased search for septic patients in the emergency services, specific Nursing studies published on recognized scientific platforms are still incipient, especially with regard to the early recognition of sepsis by nurses, from the risk classification. No studies were found relating the applicability of specific scores for monitoring and predicting clinical outcomes such as sepsis, septic shock and in-hospital mortality.

With this demand, the following guiding question arose from the researchers: “What is the performance of the quickSOFA scores and Systemic Inflammatory Response Syndrome (SIRS) as predictors of clinical outcomes in patients admitted from an emergency service?”

Therefore, the main objective of this study was to evaluate the performance of the quickSOFA and SIRS scores as predictors of clinical outcomes in patients admitted from the emergency service of a University hospital in southern Brazil.

Through this study, it is intended to qualify assistance to the users, identifying early predictors of unfavorable clinical outcomes such as sepsis, septic shock and inhospital mortality through the use of these scores, from the risk classification in these services. The incorporation of the results of this research may benefit the care and management practice in this scenario, actively contributing to the improvement of processes, protocols and workflows, especially with regard to the prevention of mortality.

## Method

The methodological report of this study was carried out according to the guidelines of the Strengthening the Reporting of Observational Studies in Epidemiology (STROBE)^(9)^.

### Study design and context

A retrospective cohort study, carried out between January 1^st^, 2017 and May 31^st^, 2018, in the Emergency Service (ES) of a Public University Hospital in Southern Brazil.

### Participants

A total of 122 patients were included according to the eligibility criteria: A) Inclusion: patients over 18 years old, cared for and hospitalized in the ES, who were welcomed and classified by the nurse in the screening room and assigned, along with the classification, the score for the application of the qSOFA score upon their arrival at the service b) Exclusion: patients without a qSOFA score completed by the nurse professional at any time in their risk classification and electronic medical records with missing data to assess the SIRS score.

### Data sources/measures

The information of the researched subjects was obtained from the hospital’s assistance database, generated through a database made available in spreadsheets in the MS Excel^®^ program. In this database, data on patients admitted to the ES in the study period were made available. After the spreadsheet was made available, the medical records were chosen at random through a draw tool within the own software used for statistical analysis where the patients included in the sample were defined during the study period. The data were obtained exclusively through the review of electronic medical records. The patients were divided in two groups: Patients WITH sepsis and patients WITHOUT sepsis. It is worth mentioning that, after obtaining the data, they were checked and typed in the Excel^®^ program by two different typists (main researcher and research assistant), being subsequently compared to control possible typing errors.

### Variables and Outcomes

The variables were classified in two groups: sociodemographic, referring to the risk classification, and clinical variables related to hospitalization. The institution where the study was carried out uses the Manchester Screening System (MSS) in the emergency service as a risk classification system. The MSS aims to identify the user’s main complaint, and to select a specific flowchart, guided by discriminators who determine service priority. The individual can be classified in five different priority levels: 1: Emergency; 2: Very Urgent; 3: Urgent; 4: Little Urgent; and 5: Not Urgent. Each priority level has its specific color and the recommended initial service time^(10)^. The variables that were part of the database referring to the patients’ risk classification were the following: flowchart used, discriminator chosen, priority of the care assigned, vital signs, and the result of the qSOFA score recorded by the nurse at the time of risk classification.

To achieve the study objectives, the values of the following scores were used: qSOFA, SIRS and Charlson’s Comorbidity Index (CCI). The qSOFA score is recorded jointly with the MSS in the risk classification by the nurses, since the SIRS and the CCI are not necessarily performed at risk classification, the first one because it does not contain all the variables necessary to obtain it, such as some results of laboratory tests; and the other, for not being able to delay the risk classification in the search for the patient’s previous comorbidities. Therefore, it is worth mentioning that the information for calculating them was extracted exclusively from the electronic medical records of the research participants. These calculations were performed by researchers trained to obtain the scores and were obtained as follows:

In the field where the study was conducted, there is a care line for septic patients, where the application of the qSOFA score is performed in the care and initial assessment by the nurses at risk classification, as part of the protocol of this problem, in addition to the application of the MSS.

The qSOFA score is considered positive for a possible diagnosis of sepsis, when showing two or more criteria (points) of patient evaluation: respiratory rate equal to or greater than 22 respiratory movements *per* minute (rmpm), change in the level of consciousness, verified through the application of the Glasgow Coma Scale <15, or systolic pressure less than or equal to 100 mmHg^(2)^.

In the event of an abnormality of this score, the system signals this patient in a different color from the others (purple), in order to signal to the medical team the need for early medical assistance to the patient with a probable diagnosis of sepsis, signaled by this score by the risk classification nurse. It is worth mentioning that all the nurses who carry out risk classification in the Emergency Service were trained by the Brazilian Risk Classification Group (*Grupo Brasileiro de Classificação de Risco*, GBCR) to apply the MSS, in order to accurately define the priority of care for patients who seek the emergency service. In addition to this, these classifying nurses received specific training to apply the qSOFA score to all patients with symptoms for sepsis.

To obtain the SIRS score, which is defined by the presence of at least two of the following signs: central temperature > 38.3ºC or <36ºC, heart rate > 90 bpm, respiratory rate > 20 rmpm, or PaCO_2_ <32 mmHg and total leukocytes > 12,000/mm³; or <4,000/mm³ or presence of > 10% of young forms (left deviation)^(1)^; the laboratory tests were verified after the first results came out, in order to properly assess this score. These exams were consulted in the electronic medical record of the selected patients along with the registration of vital signs at risk classification, as a way to complete the assessment of the score.

Finally, to obtain the CCI, which is a tool used to verify the prediction of in-hospital mortality, the MDCalc^®^ online calculator was used, which analyzes the age and the list of previous comorbidities recorded in the electronic medical record of each study participant. This calculator follows the modifications for the evaluation of the updated index foreseeing 16 comorbidities that generate different scores, the result being established by the sum of all, associated with the patient’s age. The higher this score, the lower the subject’s life expectancy in the next 10 years^(11)^. The CCI had its score value categorized as <2 (without risk) and as > 3 (with risk), in order to classify the patients› risk in relation to the presence of comorbidities for the mortality outcome.

The main outcome of this study was the diagnosis of sepsis and the secondary outcomes were septic shock and the occurrence of death due to sepsis during the patient’s in-hospital stay, recorded in medical records, and confirmed by reviewing the hospital discharge or death summary. The mortality rate was measured from hospital admission to death.

### Sample size

Sample calculation was carried out in two stages: one for the main study objective (qSOFA and SIRS *vs.* Sepsis and septic shock) and one for the secondary objective (qSOFA and SIRS *vs.* Mortality). For the first stage, the same was done in the R/R Studio^®^ program, version 3.5.3, using the pROC package and the *power. roc.test* function.

Considering the prevalence of sepsis of 30% in Brazilian and international studies^(12-13)^, power of 95% and significance level of 5%, a sample size of 50 patients is sufficient to detect as significant an area under the ROC curve of 0.7 considering qSOFA as a predictor of sepsis diagnosis and a clinically useful test to be used in the ES for this early identification.

For the second stage (qSOFA *vs.* Mortality), the sample calculation was performed with the WinPEPI program, version 11.43. Considering 80% power, 5% significance level, and the following data^(13)^: 75.2% of the patients with a qSOFA score below 2, 3.3% of mortality in patients with a qSOFA score below 2 and 23.9% of mortality in patients with a qSOFA score greater than or equal to 2, the size total sample of 122 subjects was defined. Thus, in an attempt to answer the two outcomes proposed, the sample size of the largest number of subjects will be used.

### Quantitative variables

The continuous variables were described from their means and standard deviations and the categorical variables by using frequencies and proportions. The qualitative variables, such as gender, were compared using the chisquare and Fisher’s exact tests and the continuous variables with the Student’s T and Mann-Whitney tests (according to the normality of the variable). The statistical tests were defined after performing the Kolmogorov-Smirnov test to verify the normality of the numerical data. The comparison of the characteristics between groups 1 and 2 (WITH sepsis and WITHOUT sepsis) was performed.

### Statistic methods

The data collected were organized and compiled in the Excel software and later submitted to the statistical programs Statistical Package for the Social Sciences (SPSS), version 18.0, and R, version 3.5.2. Multivariable analysis was performed using the Poisson Regression method with robust variance in order to estimate the effect of the predictive factors in relation to the occurrence of the studied outcomes. Associations with a p-value <0.05 were considered significant. The analysis of the ROC curve was performed by estimating the area under the curve (AUC) in order to estimate the accuracy of the scores (qSOFA and SIRS) in relation to the outcomes (sepsis, septic shock and mortality). Considering the cutoff points defined by the authors, diagnostic tests were verified, such as sensitivity and specificity. The confidence intervals were calculated considering the 95% confidence level.

### Ethical aspects

The research was submitted to and approved by the Ethics and Research Committee of the Institution under number 2017-0652, Certificate of Presentation for Ethical Appreciation (*Certificado de Apresentação para Apreciação Ética*, CAAE) 80987617.6,0000.5327 and opinion No. 2,455,554/2017, and is in accordance with Resolution 466/2012 of the National Health Council.

## Results

The results were divided in two stages: in the first, the socio-epidemiological profile of the septic and nonseptic patients in the study was assessed and, in the second, the univariate and multivariate analyses of the other data from the database were carried out.

A total of 122 subjects were included for the study; of these, 71 (58.2%) developed sepsis, 45 (63.4%) were male, and 62 (87.3%) were white-skinned, with mean age ± standard deviation (SD) of 62 ± 18.43 years old, with a minimum age of 18 and a maximum of 95 years old. Nearly 39 (54.9%) subjects in the sample had completed elementary school. The median length of hospital stay for patients with sepsis was 8 (4-14) days, with a maximum of 144 days and a minimum of 1 day. The mean score of the Charlson Index was 4.46 ± 2.62, with 74.6% of the septic patients having scores >3 on this index. The mortality rate of the sample was 23.8% (n=29), of which 25 (86.2%) developed sepsis, as described in Table 1.

The most prevalent infection site was the respiratory system, with 32.8% of the cases, followed by the urinary site, with 18%. Sepsis was more prevalent in patients of the sample diagnosed with infection with multiple sites, with 83.3%, followed by the abdominal wall focus with 76.9%. The prevalence of septic shock was higher in patients with abdominal wall infection, with 69.2%, followed by skin focus with 62.5%. The highest mortality rate occurred at the abdominal wall site with 53.8%, followed by the gastrointestinal tract with 50%.

**Table 1:** Description of the sample's epidemiological and demographic profile (N* = 122). Porto Alegre, RS, Brazil, 2018

Variables	Characteristic of the studied population				p^‡^-value
	Septic patients (N*)	% (CI^†^ 95%)	Non-Septic patients	% (CI^†^ 95%)	
N*	71	58.2 (48.9-67.1)	51	41.8 (32.9-51.1)	
**Demographic**					
Age, mean (±SD^§^)	62.56 (18.43)	-	56.98 (19.45)	-	0.113^||^
Male	45	63.4 (51.1-74.5)	24	47.1 (32.9-61.5)	0.118|^¶^
**Schooling**					< 0.001**
Unknown	4	5.6 (1.6-13.8)	1	2.0 (0.0-10.4)	
Incomplete Elementary School	1	1.4 (0.0-7.6)	4	7.8 (2.2-18.9)	
Complete Elementary School	39	54.9 (42.7-66.8)	29	56.9 (42.2-70.7)	
Incomplete High School	11	15.5 (8.0-26.0)	9	17.6 (8.4-30.9)	
Complete High School	2	2.8 (0.3-9.8)	2	3.9 (0.5-13.5)	
Incomplete Higher Education	14	19.7 (11.2-30.9)	5	9.8 (3.3-21.4)	
Complete Higher Education	0	-	1	2.0 (0.0-10.4)	
**Skin color**					0.579^||^
White	62	87.3 (77.3-94.0)	42	82.4 (69.1-91.6)	
Black	8	11.3 (5.0-21.0)	6	11.8 (0.4-23.9)	
Brown	1	1.4 (0.0-7.6)	2	3.9 (0.5-13.5)	
Not declared	0	-	1	2.0 (0.0-10.4)	
Length of stay	8 (4-14)	-	11 (6-19)	-	0.217**
Mean Charlson score	4.46 (2.62)	-	4.5 (2.30)	-	0.92^||^
					0.73^¶^
2≤	18	25.4 (15.8-37.1)	8	15.7 (7.0-28.6)	
> 3	53	74.6 (62.9-84.2)	43	84.3 (71.4-93.0)	
Deaths	25	35.2 (24.2-47.5)	4	7.8 (2.2-18.9)	

* N = Number of cases;

† CI = Confidence interval;

‡ p = Significance level;

§ SD = Standard deviation;

|| Student's t test for independent samples;

¶ Pearson's chi-square test;

** Mann-Whitney test

As for the qSOFA score, 55.7% of the patients had qSOFA <2, of which 35.3% developed sepsis, with 8.2% of the sample progressing to septic shock and 11.8% evolving to death. Among the total sample, 44.3% had qSOFA >2, of which 87% developed sepsis, 55.6% progressed to septic shock and 38.9% evolved to death.

Of the three criteria evaluated by qSOFA, the one with the highest number of changes was respiratory rate greater than 22 rmpm (52.4%), whereas the presence of sepsis, septic shock and death was more prevalent in patients with altered level of consciousness in the Glasgow Coma Scale with 84.8%, 57.6% and 45.5%, respectively.

As for the SIRS score, 78.51% of the patients obtained SIRS >2; of these, 66.3% developed sepsis, 40% of the patients in the sample evolved to septic shock and 29.5%, to death.

The highest probability of developing septic shock was due to abdominal wall focus (RR: 5.07; CI_95%_ = 1.66 - 15.44; p<0.004), followed by skin focus (RR: 4.58; CI_95%_ = 1.40 - 14.92; p<0.011). In relation to death, there was a greater chance also in those due to abdominal wall focus (RR: 5.92; CI_95%_ = 1.44 - 24.35;p <0.05) and the gastrointestinal tract focus (RR: 5.50; CI_95%_ = 1.23 - 24.45; p<0.05).

QSOFA ≥2 was associated with hospital death (RR: 3.30; CI_95%_ = 1.59 - 6.87; p<0.001), with sepsis (RR: 2.46; CI_95%_ = 1.75 - 3.45; p<0.001), and with septic shock (RR: 3.77; CI_95%_ = 2.03 - 7.02; p<0.001). SIRS >2 was associated with the development of sepsis (RR: 1,916; CI_95%_ = 1.10 - 3.31; p<0.004) and of septic shock (RR: 3.46^; CI^_95%_ = 1.16 - 10.33; p<0.005).

The reduction in the Glasgow score and the presence of hypotension were related to a higher probability of developing sepsis (RR: 1.75; CI_95%_ = 1.35 - 2.27; p<0.001) (RR: 1.786; CI_95%_ = 1.32 - 2.41; p<0.001) and septic shock (RR: 2.44; CI_95%_ = 1.51 - 3.93; p<0.001) (RR: 2.13; CI_95%_ = 1.26 - 3.57; p<0.004) and death (RR: 2.89; CI_95%_ = 1.57 - 5.31; p<0.001) (RR: 2.32; CI_95%_ = 1.20 - 4.48; p<0.012). The data reported above are shown in Table 2.

**Table 2:** Clinical characteristics related to the diagnosis of sepsis x qSOFA* and SIRS† (N‡ = 122). Porto Alegre, RS, Brazil, 2018

Characteristics	N^‡^ (%)	Sepsis (%)	Septic Shock (%)	Death
**Infection Site**				
Pulmonary	40 (32.8)	24 (60.0)	12 (30.0)	10 (25.0)
Urinary	22 (18.0)	12 (54.5)	3 (13.6)	2 (9.1)
Abdominal wall	13 (10.7)	10 (76.9)	9 (69.2)^§^	7 (53.8)^§^
Cutaneous	8 (6.6)	6 (75.0)	5 (62.5)^§^	3 (37.5)
Gastrointestinal tract	8 (6.6)	4 (50.0)	3 (37.5)	4 (50.0)^§^
Others	19 (15.5)	10 (52.6)	5 (26.3)	2 (10.5)
Multiple	6 (4.9)	5 (83.3)	3 (50.0)	1 (16.7)
**qSOFA** *				
0	21 (17.2)	8 (38.1)	2 (9.5)	0 (0.0)
1	47 (38.5)	16 (34.0)	8 (17.0)	8 (17.0)
2	45 (36.9)	39 (86.7)	24 (53.3)	16 (35.6)
3	9 (7.4)	8 (88.9)	6 (66.7)	5 (55.6)
qSOFA *≥2				
No	68 (55.7)	24 (35.3)	10 (8.2)	8 (11.8)
Yes	54 (44.3)	47 (87.0)^§^	30 (55.6)^^§^^	21 (38.9)^^§^^
**Reduction in Glasgow Score**	33 (27.0)	28 (84.8)^§^	19 (57.6)^§^	15 (45.5)^§^
**Respiratory Rate>22**	64 (52.4)	39 (60.9)	26 (40.6)	18 (28.1)
**Systolic Blood Pressure<100**	50 (41.3)	39 (78.0)^§^	24 (48.0)^^§^^	18 (36.0)^§^
**SIRS^†^**				
1	5 (4.13)	1 (20)	0 (0)	0 (0)
2	21 (17.35)	8 (38.1)	3 (14.3)	3 (14.3)
3	43 (35.53)	22 (51.1)	12 (27.9)	10 (23.2)
4	39 (32.23)	31(79.5)	20 (51.3)	13 (33.3)
	13 (10.74)	10 (76.0)	6 (46.1)	5 (38.5)
**SIRS^†^** **(>2 points)**				
Yes	95 (78.51)	63 (66.3)^§^	38 (40.0)^^§^^	28(29.5)

* qSOFA = quickSOFA;

† SIRS = Systemic Inflammatory Response Syndrome;

‡ N = Number of cases;

§ Statistically significant results - using the chi-square test with 5% significance level

**Figure 1:**
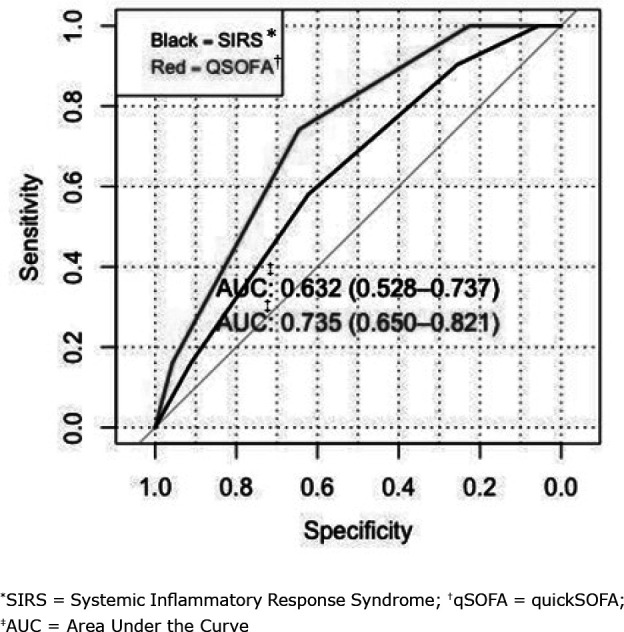
ROC curve of the qSOFA and SIRS scores in relation to sepsis

To predict the diagnosis of sepsis, qSOFA ≥2 obtained a sensitivity of 66% (CI_95%_ = 53 - 76) and a specificity of 86% (CI_95%_ = 73 - 93). The strength of the qSOFA prognostic accuracy for sepsis was confirmed with an AUC of 0.735 (CI_95%_ = 0.65 - 0.82). The SIRS score had a sensitivity of 87.5% (CI_95%_ = 80 - 95) and a specificity of 34.7% (CI_95%_ = 21 - 48). The strength of the SIRS prognostic accuracy for sepsis was an AUC of 0.632 (CI_95%_ = 0.53 - 0.74), as shown in Figure 1. When comparing the performances of SIRS and qSOFA for sepsis prognosis, no statistical difference was obtained (p = 0.3327).

**Figure 2:**
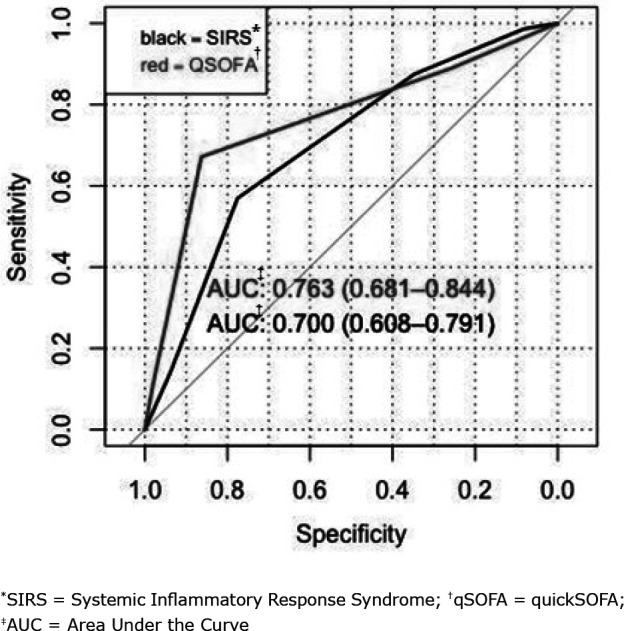
ROC curve of the qSOFA and SIRS scores in relation to sepsis septic shock

To predict the diagnosis of septic shock, qSOFA ≥ 2 obtained a sensitivity of 76% (CI_95%_ = 63.3 - 76) and a specificity of 70% (CI_95%_ = 59 - 79). The strength of the qSOFA prognostic accuracy for septic shock was confirmed with an AUC of 0.75 (CI_95%_ = 0.67 - 0.84). The SIRS score obtained sensitivity and specificity values of 92.7% (CI_95%_ = 84.7 - 100) and 29% (CI_95%_ = 18.8 - 38.7), respectively. The strength of the SIRS prognostic accuracy for septic shock was confirmed with an AUC of 0.68 (CI_95%_ = 0.59 - 0.77), as shown in Figure 2.

When comparing the performances of SIRS and qSOFA for septic shock prognosis, no statistical difference was obtained (p = 0.22).

**Figure 3:**
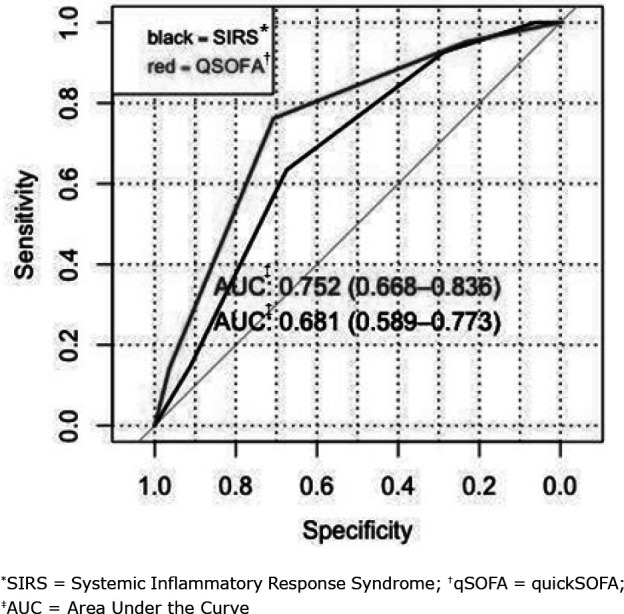
ROC curve of the qSOFA and SIRS scores in relation to death

To predict the diagnosis of Death, qSOFA ≥2 obtained a sensitivity of 72% (CI_95%_ = 52 - 76) and a specificity of 64% (CI_95%_ = 53 - 73). The strength of the qSOFA prognostic accuracy for Death was confirmed with an AUC of 0.763 (CI_95%_ = 0.68 - 0.84). The SIRS score had a sensitivity of 90.3% (CI_95%_ = 80 - 100) and a specificity of 25.6% (CI_95%_ = 16.5 - 34.6). The strength of the SIRS prognostic accuracy for septic shock was confirmed with an AUC of 0.70 (CI_95%_ = 0.61 - 0.79), as shown in Figure 3. When comparing the performances of SIRS and qSOFA for sepsis prognosis, no statistical difference was obtained (p = 0.085).

## Discussion

The original methodology provided by the new definitions of Sepsis-3 was applied to a database in a hospital of southern Brazil, in which it was found that more than half of the patients suspected of infection in the emergency service in which the qSOFA and SIRS scores were applied were diagnosed with positive sepsis. In this study, qSOFA showed better results in relation to the SIRS score in the emergency service. qSOFA ≥2 obtained higher specificity for the diagnosis of sepsis, as well as better specificity for septic shock and specificity for mortality, whereas the SIRS showed better results for sensitivity in the diagnosis of sepsis, septic shock and in-hospital death.

When comparing these results with previously conducted studies, a recently published systematic review with meta-analysis was found that related the diagnosis of sepsis to qSOFA and SIRS, where it described the specificities of these scores for the diagnosis of sepsis and in-hospital mortality. In this research, some articles revealed that, when comparing qSOFA with SIRS, it was shown that SIRS was more sensitive and significantly superior to qSOFA for diagnosing sepsis, but qSOFA was better for predicting hospital mortality. Current articles also show higher in-hospital mortality within 30 days for patients with qSOFA >2^(14)^. When compared to the performance of these scores for the prognosis of sepsis and death, our study did not show any statistical difference^(15)^.

Similarly to other analyses, qSOFA ≥2 and SIRS >2 were related to a higher number of deaths in relation to patients whose scores were below two^(16)^. In addition to that, there was also higher sensitivity for predicting mortality for the SIRS score; however, specificity was higher in qSOFA, as already shown in previous research studies^(16)^.

As in other published studies, the most common infection site was the respiratory system, followed by the urinary site^(15,17)^. However, when related to death, our research showed that the abdominal wall focus and gastrointestinal tract focus sites had a higher chance of mortality.

It should be noted that there are few Brazilian studies on sepsis and almost none published in the South of the country, with little knowledge of the profile of this population and the clinical characteristics of this disease in Brazil. The profile of the patient admitted to the emergency service due to suspected sepsis, in most of the findings in other studies, describes results similar to those of this study: mean age between 60 and 70 years old, with little difference between the genders of these patients (63% male) and presence of previous comorbidities (which we demonstrated through the CCI), in addition to a hospital stay close to 10 days^(15,18-19)^. Thus, we can characterize this sample as a population that is already aged, with previous comorbidities, and not having a great distinction regarding gender. It is important to show that, when compared to the patients who did not develop sepsis, the septic patient has higher mortality rates.

The area under the ROC curve (AUC) summarizes in a good manner the overall accuracy of a test, as it summarizes sensitivity and specificity. The area ranges from 0.5 for a useless test to 1.0 for a perfect test. Tests without discriminatory power have an area of 0.5, while values above 0.8 indicate an excellent test, and 0.7-0.8 show that the test is clinically useful^(20)^. The AUC is a measure of the overall performance of a diagnostic test, and should be interpreted as the mean sensitivity value for all possible specificity values. Considering that the AUC is a measure of the overall performance of a diagnostic test, the performance of two different tests can be compared by comparing their AUCs. As previously mentioned, the higher the AUC, the better the test to be applied in the referred context. Apparently, qSOFA served as a practical score and with good results for use in the emergency service, being a clinically useful test according to results of area under the ROC curve (AUC). Due to the need for a quick initial assessment in the reception of patients in emergency services, SIRS proved to be a less effective score for use in the emergency service; in addition, it did not prove to be a useful test according to the results of the area under the ROC curve (AUC) for the diagnosis of sepsis.

In this study, the strength of the prognostic accuracy of qSOFA and SIRS for in-hospital mortality was confirmed with an AUC of 0.73 (CI_95%_ = 0.63 - 0.83) and of 0.70 (CI_95%_ = 0.61 - 0.79), respectively, comparing to other authors who showed values close to ours^(17-18)^. No data were found in the literature that mentioned the area under the ROC curve (AUC) verifying the strength of the prognostic accuracy of qSOFA and SIRS for sepsis and septic shock from risk classifications.

However, the discussion of which would be the best tool is still brought up on a large scale. Almost all the articles chosen to discuss our research contribute a comparison between qSOFA and other tools such as SIRS, SOFA and the Modified Early Warning Scores (MEWS). There is disagreement as to the most appropriate tool to be used in the emergency service; some argue that qSOFA is very restricted and thus ends up not being able to capture all septic patients, with the use of SIRS being better^(18,21-22)^. However, there are studies that address the ease of the qSOFA tool as an early detector because it is simple to apply and does not require laboratory tests to be performed^(12,23)^. Other studies discuss the combination of the two scores, SIRS and qSOFA, as a method to improve the prognosis and detection of patients who come to the hospital due to some infection, but not applied by nurses at risk classification in patients admitted from emergency services^(24)^. There are also some articles that contribute positive and negative points of each tool, not reaching consensus on which one should be used^(13,16)^.

It is necessary to mention that qSOFA was not developed with the purpose of diagnosing sepsis, but rather as an alert tool so that an early assessment can be carried out on the patient who seeks the emergency service and shows possible signs of infection with a risk of early deterioration of their clinical condition. However, as it is a recently discovered score, research studies about its potentialities must be carried out in order to determine the best use of this tool in the initial clinical evaluation of these patients. To this end, this study showed that the nurse’s performance at risk classification is completely feasible for the early identification of possibly septic patients.

The non-comparison with other existing scores, such as MEWS, to analyze other possibilities and their performances in the emergency service with the aim of early identifying the patient in the front line of care can be considered a study limitation.

## Conclusion

In this retrospective cohort study, we found that qSOFA ≥2 had higher specificity for the diagnosis of sepsis as well as improved specificity for septic shock and specificity for mortality; on the other hand, SIRS showed better statistical results for sensitivity in the diagnosis of sepsis, septic shock and hospital death. In addition to that, we were able to characterize the sample as an aged population, with previous comorbidities, and not having a great distinction as to gender. We also emphasize that qSOFA served as a better score due to its practicality and good results for clinical use in the emergency service, as it resulted in greater prognostic accuracy for in-hospital mortality; however, we warn against the need for new prospective studies that cover other tools so as to identify which would be the most accurate and with the best performance and clinical applicability in this scenario.
